# Experimental investigation of surface roughness effects on energy harvesting from a piezoelectric eel behind a cylindrical bluff body

**DOI:** 10.1371/journal.pone.0327916

**Published:** 2025-07-17

**Authors:** Muhammad Hammad Bucha, Niaz Bahadur Khan, Emad Uddin, Hafiz Hamza Riaz, Adnan Munir, Umar Farooq, Ming Zhao, Riaz Muhammad, Mohammed Jameel, Muhammad Tauseef Nasir, M. Nafees Mumtaz Qadri

**Affiliations:** 1 School of Mechanical & Manufacturing Engineering (SMME), National University of Sciences and Technology (NUST), Islamabad, Pakistan; 2 Mechanical Engineering Department, College of Engineering, University of Bahrain, Isa Town, Bahrain; 3 Department of Mechanical and Computer-Aided Engineering, National Formosa University, Yunlin 632, Taiwan, Republic of China; 4 School of Engineering, Design and Built Environment, Western Sydney University, Penrith, New South Wales, Australia; 5 Department of Civil Engineering, College of Engineering, King Khalid University, P.O. Box: 960, Asir, Abha, 61421, Saudi Arabia; NED University of Engineering and Technology, PAKISTAN

## Abstract

Due to dwindling energy reserves and the cost-effectiveness of installation, the global trajectory is shifting towards renewable energy sources as a proficient means of energy acquisition. Among these sources, hydropower stands out as it harnesses the kinetic energy of oceanic water flow to generate power. Various studies have harnessed vortex-induced vibrations (VIV) to generate power from a piezoelectric eel, showcasing the diverse applications of this technology. The present experimental study further explores this technology and investigates the effect of surface roughness of cylindrical bluff body on the energy harvested by piezoelectric eel using a low-speed water tunnel. The experiments were performed at four different roughness values (K_s_/D) namely 2.21, 4.07, 9.85, and 13.97 microns for the cylinders with diameters of 25, 27, 27.2, and 27.5 mm, respectively. The Reynolds number in the present study is fixed at 8690. A total of hundred case studies were performed to analyze the effect of the surface roughness of the cylinder on energy harvesting performance from the eel. The flapping frequency, amplitude, and optimal power of the rough cylinders were analyzed and compared with that of smooth cylinders experimentally, and the optimum point ($G_{x}=1.25,\;G_{y}=0$) in terms of power was attained. Increased surface roughness significantly reduced power output, flapping frequency, and amplitude. The smoothest cylinder (K_s_/D = $8.8\times 10^{-5}$) produced the highest power (52.325 µW), while the roughest (K_s_/D = $5.08\times 10^{-4}$) resulted in a 6.26% decrease in power (36.4 µW), along with reductions of 4.5% in flapping frequency and 20% in amplitude. By increasing the surface roughness of the bluff body, the lock-in region decreases and as a result, the harvested power from that bluff body is reduced. Moreover, the power also decreased by increasing the distance between the cylinder and eel both in the x- and y-direction. The results of the current study provide deeper insights into the effect of surface roughness on energy harvesting from piezoelectric eel behind cylindrical bluff body, that are essential for the development of efficient energy harvesting systems. The findings of this study would be useful for the design of piezoelectric eel-based energy harvesting devices in marine environments.

## Introduction

The world is moving towards renewable energy resources as an efficient way of energy harvesting because of the depleting energy resources and the low installation cost. One of the sources of energy harvesting is hydel power, which harnesses the flow of water such as in oceans and rivers to generate electricity [1–3]. Among emerging technologies, one method utilizes the piezoelectric effect to generate electricity from fluid flow interacting with a simple bluff body [4,5]. Recent advances have focused on enhancing piezoelectric energy harvesters, such as using double cantilever beams for coupled bending-torsion vibrations [6], improving efficiency through novel materials and structural designs [7], and developing intelligent elastic devices [8] In particular, recent theoretical studies have demonstrated performance benefits of fluid-immersed piezoelectric microstructures under coupled thermal and magnetic environments using advanced materials such as graphene-reinforced composites [9]. Piezoelectric materials are widely used in energy harvesting for wearable electronics, IoT devices, and strain gauges [10]. This small-scale energy harvesting plays a crucial role in powering portable devices such as laptops, surveillance recorders, and self-sustaining power supplies [11].

**Fig 1:**
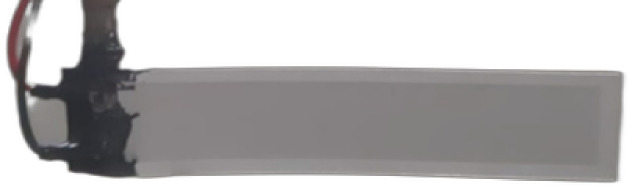
Piezoelectric eel.

Piezoelectric energy harvesting systems utilize the piezoelectric effect, where mechanical stress induces an electrical charge in a piezoelectric material, enabling the conversion of kinetic energy into electrical energy, which is ideal for small-scale, low-power applications. The system typically consists of a flexible piezoelectric material that deforms under mechanical stress, generating voltage in response to various stimuli such as fluid flow, vibrations, or motion. A key mechanism in this process is the interaction between a bluff body, like a cylinder or eel-shaped structure, and fluid flow, which generates vortex-induced vibrations (VIV). As vortices form behind the bluff body, they induce oscillations that can be harnessed by piezoelectric materials to generate energy. The efficiency of this process depends on factors such as the bluff body’s geometry, the spacing between the bluff body and the piezoelectric material, and the flow characteristics, including the Reynolds number [12]. Optimal spacing (S/D ratio) enhances vortex shedding and improves energy capture, while variations in flow speed and Reynolds number influence vortex dynamics and energy output. The bluff body induces vortices in the flow, which causes stress in a piezoelectric material, usually in the shape of thin, flexible membranes or eels, and the material, in turn, generates energy [13,14]. Fig 1 shows a piezoelectric eel, which when placed in the flow domains of rivers, streams, and oceans, can extract energy to power small systems without severely damaging the ecosystem. Eels placed axially in the direction of flow generate energy by flapping. This is due to the interaction with induced vortices [15]. Energy harvested using piezoelectric eels has many modern low-input power applications. Underwater microelectromechanical devices used as sensors and actuators [16], especially in offshore oil and gas extraction sites, can be made self-sufficient with the use of this technology [17].

Fig 1 shows a piezoelectric eel connected to one end with metal support and an electrical connection. In such a configuration, the eel’s flapping resembles the vibration of a cantilever beam. The flapping area of the eel plays a significant role in terms of energy outputs. The area of eel is compared in terms of ratio of spacing between the bluff body and eel to diameter of the bluff body (S/D), ratio of length of piezoelectric eel to diameter of the bluff body (L/D), and the dynamic variation of Reynolds number. To visualize the variation in results, experimental data sets are organized based on different diameters. The speed of flowing water is also varied to collect different data sets. Sub-critical points and post-critical points are considered as well for precise results. In upstream flow, moving towards the flow enhances energy harvesting due to increased flapping, making the process more effective. As the S/D ratio increases from 1 to 2, a significant rise in voltage is observed. However, when S/D reaches 3, vortex shedding diminishes, leading to a drop in voltage. The points of maximum energy gain were analyzed at optimal spacing and flow speed (U) to achieve the highest energy output.

Allen and Smits [18] investigated the potential of generating power in the ocean through the flexible piezoelectric membranes which were excited by the vortex street producing behind a bluff body. A significant increase in energy was observed in the study conducted by Latif, et al. [19] and an increase of 38% in energy generation was reported by changing the S/D ratio of the experimental setup. Mujtaba, et al. [20] investigated the energy harvested from an inverted C-type cylinder positioned at flow regions with cut angles of 120, 150, and 180 degrees. They observed an increase in energy output by up to 38.70%, 46.70%, and 43.40%, respectively, compared to a circular cross-section when the cylinder was placed on the middle line. The wakes generated by the inverted C-type cylinders varied depending on their types, resulting in differences in the location of energy maxima. Latif, et al. [21] performed a comparative analysis of bluff body-induced wake effects on a piezoelectric plate, demonstrating up to 847% improvement in energy harvesting output using optimized bluff body geometries. Ding, et al. [22] investigated the oscillations generated by a spring-mounted circular cylinder in a wind tunnel at high Reynolds numbers. They reported flows close to the critical Reynolds number and applied even roughness to the cylinder using glass-bead minor particles. During the free oscillation test, the velocity was decreased from 5 to 8 m/s, and mass damping was maintained up to 6 m/s. The relative low speed of the wind was kept close to the critical Reynolds number when the cylinder’s surface roughness was applied. They found that negative damping was too small near the critical Reynolds number, which could be improved by adding small structural damping close to the critical Reynolds number. Allen and Henning [23] investigated the induced vortices for cylinders with surface roughness ranging from $5.1\times 10^{-5}$ to $5.8\times 10^{-3}$ and Reynolds numbers ranging from $1.8\times 10^{5}$ to $6.5\times 10^{5}$. A significant impact of the variation in Reynolds number on the ratio of convective forces to diffusive forces was reported for a rough circular cylinder. They identified the drag and VIV at critical and subcritical points of the flexible cylinder, up to 20 diameters upstream. They further highlighted that less VIV results in lower frequency and reduced harvested energy. Additionally, the roughness factor (K_s_/D) was identified as an important factor in checking the effect of roughness on the bluff body and for making dimensionless studies. Bernitsas, et al. [24] investigated the impact of Reynolds numbers ranging from $8\times 10^{3}$ to $2\times 10^{5}$ and surface roughness ranging from $1.4\times 10^{-5}$ to $4.2\times 10^{-3}$ on VIV behavior. It was found that the roughness of the cylindrical bluff body has a strong impact on flow transition, separation point, drag, and correlation length in VIV. They noted that roughness is correlated to the suppression and compression of VIV by varying the starting, ending, and length of the base support. The roughness size was defined in terms of the grit size of the surface of the bluff body, which cannot be zero in practical life. It was also found that increasing the roughness decreased the harvested energy because the disturbance produced by the bluff body amplified the vortex frequency and increased the transition range. As a result, less power is harnessed due to the narrower shear layer of the wake and immediate dissipation of the formed vortex.

In their numerical study, Gao, et al. [25] investigated the VIV response characteristics of cylindrical bodies with varying surface roughness. Three different values of roughness were used, and the regions were divided into four regimes (1, 2, 3, and 4), with regimes 2 and 3 belonging to the lock-in region. Factors such as vortex shedding, lock-in region, vortex shedding frequency, upper branch, desynchronization zone, and lower branch were considered. The results showed that the VIV response decreased with an increase in the roughness factor. The findings also demonstrated that as the roughness increased, the lock-in region became narrower, the Strouhal number (Vr) increased, and the drag coefficient showed a downward trend. It is interesting to note how the surface roughness of a cylinder can affect its wake vortex shedding behavior. The wake vortex shedding region of a smooth cylinder is different from that of a rough cylinder due to several factors such as the direction of traversal, phase difference of traverse vibrations, vibration amplitude, and lift force. When the surface roughness is increased from a smooth cylinder to a rough cylinder, it tends to suppress the formation of the wake of 2P vortex shedding, which alters the pattern of the wake of the 2S vortex. This finding was reported by Han, et al. [26]. In addition, it was observed that at low reduced velocities, the range of chaotic motion and multiple frequencies for a rough cylinder reduced as compared to a smooth cylinder. However, at high reduced velocities, the range of chaotic motion for a smooth cylinder tends to increase compared to a rough cylinder. These observations demonstrated how the surface roughness of a cylinder can significantly affect its wake vortex shedding behavior and highlight the importance of understanding the impact of surface roughness in studies related to VIV of cylindrical bluff bodies.

Ghazali, et al. [27] conducted an experimental study to verify VIV for surfaces with different roughness factors. After performing free vibration tests, the response of the smooth cylinder was recorded at a reduced speed. A variety of roughness levels were tested in the experiment. The experiment’s conclusion showed that as roughness increased, the amplitude response of the vibrating bluff body decreased, and the wake region became narrower in the low reduced velocity region. Ramzi, et al. [28] conducted a simulation study on cylinders with varying roughness. The study investigated the impact of surface roughness on vortex-induced vibrations in detail, showing that higher roughness led to greater reductions in amplitude. The researchers focused on the effect of low reduced velocity on a short rigid cylinder and found that increasing roughness caused the lock-in region to become narrower and closer. They also observed an increase in amplitude when the frequency approached the natural frequency of 1 at low reduced velocity. Additionally, the study highlighted the constructive phenomenon of frequency matching, which resulted in high frequency and amplitude of the flapping flag. Okajima, et al. [29] conducted an experimental study on the impact of surface roughness by simulating the cylinder using a range of Reynolds numbers of $2.5\times 10^{4}$ to $3.2\times 10^{5}$ and roughness range of $5\times 10^{-3}$ to $3.8\times 10^{-2}$. A free oscillation test was carried out in a wind tunnel, including a reduced velocity range of 1.5 to 8 and a mass damping constant reduction of 6. Results showed that at Reynolds numbers higher than the critical point, a rougher cylinder had a larger amplitude of vibration. The rough cylinder generated more vibrations, resulting in the formation of chaotic flow which was more prone to dissipate with the formation of weak vortices that immediately shed off. Kiu, et al. [30] studied the range of Reynolds number ranging from $1.7\times 10^{4}$ to $8.3\times 10^{4}$ and surface roughness ranging from $2.8\times 10^{-4}$ to $1.4\times 10^{-2}$. The results of the experiment showed that traverse amplitude tended to decrease as the roughness of the cylinder decreased, which caused the narrower wake region to form. The maximum drag force also decreased as the roughness decreased. The width of the lock-in region tended to decrease as the roughness increased. Park, et al. [31] studied the varying effects of roughness strips and the Reynolds number to note their impact on shear layers. They observed that turbulence was induced in the flow by adding roughness, which weakened the vorticity and caused diffusion of the vortex. As a result of the roughness effect, the amplitude of vibration approached negligible values. The shear layer rolled up and the vortex shedding became irregular, resulting in sharp changes. The shear layer became chaotic and diffused earlier in the formation process. However, the shear layer did not impart the energy of water to the harvesting flag. Gao, et al. [32] conducted tests in a range of Reynolds numbers from $2.5\times 10^{4}$ to $1.8\times 10^{5}$ and roughness ranging from $1.1\times 10^{-4}$ to $1.2\times 10^{-2}$. The experimental results showed that the number of participating amplitudes and frequencies decreased with an increase in roughness. The onset of the lock-in region was observed in the smooth cylinder, while in the rough cylinder, the lock-in region tended to decrease abruptly. Melchers [33] investigated the behavior of aluminum in seawater, taking into consideration the development of pit size and loss of mass. While aluminum is corrosion-resistant over time, the presence of minerals in seawater causes it to corrode and erode. The researchers observed vast amounts of data for three years through experimentation, recording the depth of the pit and the loss of mass due to erosion. The amount of energy extracted depends on the strength of vortices induced in the flow, which, in turn, is influenced by the shape of the bluff body and its surface roughness. Much research has been done on cylindrical bluff bodies, whether full or cut, square bodies, and fins [34]. However, very little work has been done on the effect of bluff body aging on the energy harvested from piezoelectric eel. The current study investigates this parameter by varying the surface roughness of the cylindrical bluff body and finding the optimum location at which the harvested energy is maximum.

The different ranges of Reynolds number and surface roughness investigated in literature are listed in table 1.

**Table 1:** Studies by researchers with varying Reynolds number and surface roughness.

Literature	Reynolds Number	Surface Roughness
**Allen and Henning [23]**	$1.8\times 10^{5}-6.5\times 10^{5}$	$5.1\times 10^{-5}-5.8\times 10^{-3}$
**Bernitsas, et al. [24]**	$8\times 10^{3}-2\times 10^{5}$	$1.4\times 10^{-5}-4.2\times 10^{-3}$
**Han, et al. [26]**	$1.5\times 10^{3}-10.5\times 10^{3}$	$1\times 10^{-4}-2\times 10^{-2}$
**Okajima, et al. [29]**	$2.5\times 10^{4}-3.2\times 10^{5}$	$5\times 10^{-3}-3.8\times 10^{-2}$
**Kiu, et al. [30]**	$1.7\times 10^{4}-8.3\times 10^{4}$	$2.8\times 10^{-4}-1.4\times 10^{-2}$
**Gao, et al. [32]**	$2.5\times 10^{4}-1.8\times 10^{5}$	$1.1\times 10^{-4}-1.2\times 10^{-2}$
**Gao, et al. [35]**	$5\times 10^{3}$	$0-2\times 10^{-2}$
**Chen, et al. [36]**	$3.9\times 10^{3}$	$2.5\times 10^{-3}-\;1\times 10^{-2}$

## Experimental setup and methodology

**Fig 2:**
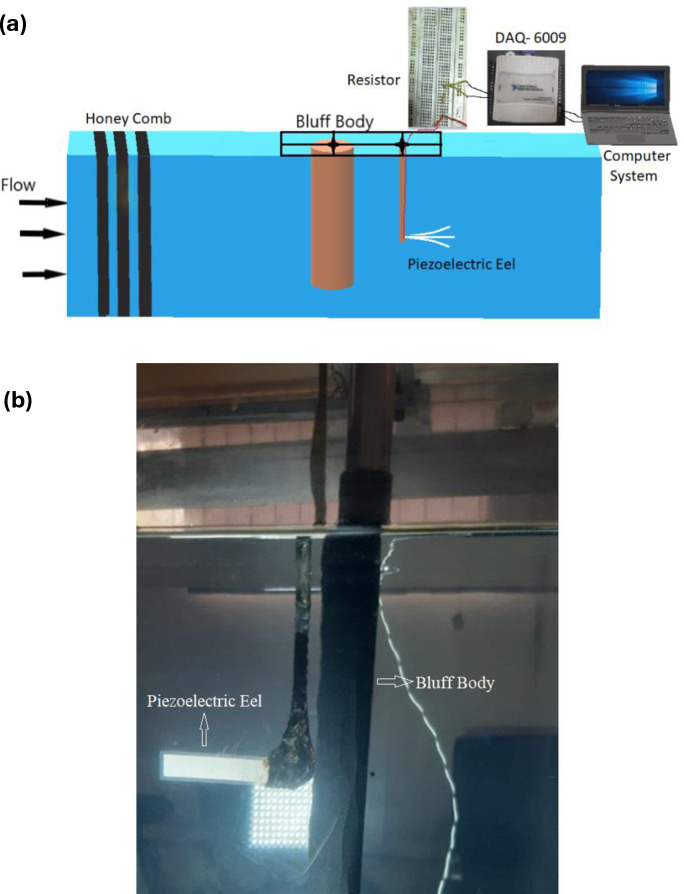
(a) Schematics of water tunnel and (b) Experimental setup.

This study was conducted in the Flow Visualization Laboratory of the Department of Mechanical and Manufacturing Engineering at the School of Mechanical and Manufacturing Engineering (SMME), National University of Sciences and Technology (NUST), and the details can be looked into from the studies [29,37]. The schematic diagram of the experimental setup is shown in Fig 2. An automated water tunnel with a water speed controller was employed, and purified water with a sediment value of less than 25 ppm was used in the experimentation.

**Fig 3:**
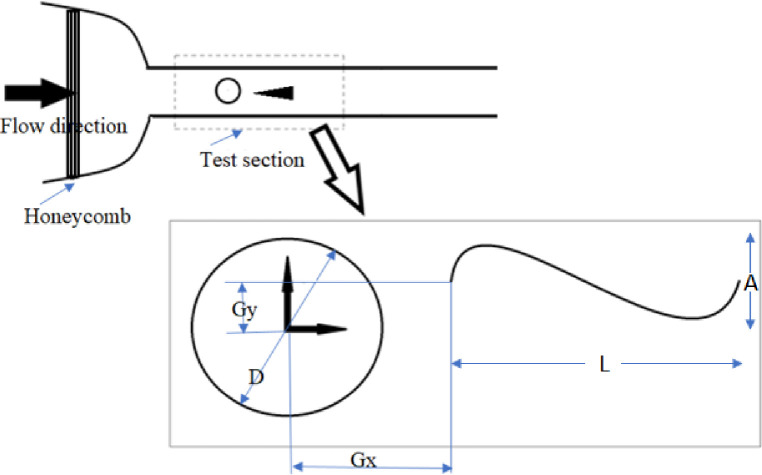
Top view of the experimental setup.

The Reynolds number was selected based on experimental constraints. A flow velocity of 0.31 m/s was maintained in the test section, resulting in a Reynolds number of approximately 8680 for cylinder diameters ranging from 25 mm to 27.5 mm. Four different cylinders were used, corresponding to a maximum blockage ratio of 6.3%, which is well below the commonly accepted threshold of 10%. The honeycomb structure was thoroughly cleaned to establish a unidirectional flow tunnel. The incoming water was directed into the tunnel through an inlet valve, and the flow was stabilized at a particular level by adjusting the rotational speed of the pump and frequency of the motor. The size of the honeycomb maze and cleanliness were verified to ensure that the flow remained laminar. The schematic shown in Fig 3 illustrates the experimental setup to study vortex-induced vibrations (VIV) of a piezoelectric eel placed downstream of a circular cylinder. The flow enters through a honeycomb structure to ensure uniformity before reaching the test section, where a circular cylinder (diameter D) generates vortices that drive eel oscillations. The eel modeled using a flexible PVDF sheet (LDT1–028K with lead attachments from Measurement Specialties, Inc.), vibrates with an amplitude A, influenced by the vortex shedding from the cylinder. The eel has a length L = 62 mm and a width H = 12 mm, with its detailed material properties listed in Table 2. The parameters $G_{x}$ and $G_{y}$ define the horizontal and vertical streamwise gaps between the eel and the cylinder (bluff body).

**Table 2:** Parameters of energy harvesting.

Optimal Resistance	1MΩ
Thickness	28 µm
Effective length	64 mm
Width	12 mm
Poison Ratio	0.46
Reynolds number	8681
Young’s modulus	1.38 GPa
Density	1.75 × 10^3^ kg/m^3^
Voltage generated	0.01-100 mV
Diameters of cylinders	1. 25 mm
	2. 27 mm
	3. 27.2 mm
	4. 27.5 mm

The experiment was conducted in a water tunnel with a velocity of U = 0.31 m/s and a pump speed of 25 hertz. The piezoelectric eel was attached to a thin steel rod with a diameter of 4 mm, and the rods were securely fastened in an aluminium lightweight clamp over the walls of the test section. The piezoelectric eel was placed in a conventional arrangement, clamped at one leading edge and free at the trailing edge, in a uniform flow immediately after the bluff body. The streamwise gaps, $G_{x}$ and $G_{y}$, between the eel and the bluff body were adjusted using an overhead mounting mechanism, as shown in Fig 3. The x-direction gap ($G_{x}$), measured from the center of the cylinder to the upstream location of the eel, ranged from 1 to 3.5, with increments of 0.5 for each cylinder roughness. Similarly, the y-direction gap ($G_{y}$) varied from 0 to 1.5, also with increments of 0.5 for each cylinder roughness. A circular aluminium cylinder was used as the bluff body, and the roughness was varied by using suitable sandpaper. The smooth cylinder had a roughness of 2.21 microns and a diameter of 25 mm. The second cylinder had a roughness value of 4.07 microns, a diameter of 27 mm, and the third cylinder had a roughness of 9.85 microns with a diameter of 27.2 mm. The final cylinder had a roughness of 13.97 microns with a coarser grit size and a diameter of 27.5 mm. To account for the effect of increasing diameters with varying surface roughness, a dimensionless parameter (K_s_/D) was utilized, where each roughness value was normalized by its respective diameter. Four different roughness values were considered, expressed as K_s_/D: $8.8\times 10^{-5},\;1.51\times 10^{-4},\;3.62\times 10^{-4},\;$and $5.08\times 10^{-4}$. The specifications of the parameters utilized in the experimentation are mentioned in Table 2.

A high-resolution and high-speed camera of the model (Sony Cyber-shot DSC-RX100 IV, fixed under the test portion) was utilized to record videos of piezoelectric eels to determine the flapping pattern at 50 frames per second for two minutes at 20.1 megapixels. A flashlight was used for the illumination of the test section. The eel was connected to a DAQ card to store the electrical signal data acquired from flapping. The voltage generated from the piezoelectric eel by applying a resistance that was connected between an eel and the DAQ card was measured. A 1 MΩ resistance was used to calculate the optimal power output by using the maximum power transfer theorem expressed by P = V^2^/R_opt_ where, P is the rate of change of energy with time (Power), V is the instantaneous voltage, and R is the optimum resistance for the relevant circuit. Moreover, the output was measured against different values of the optimal resistance to validate the similar value of 1MΩ. LabVIEW® software was used to visualize the data for further analysis of the generated voltage, with the waveform exported as an Excel sheet after post-processing. The data corresponds to the same duration as the videos, enabling amplitude and frequency analysis in MATLAB. The image processing technique was utilized to comprehend the tail position of the flag to evaluate the peak of amplitude (A/L), and the Fast Fourier Transform Method (FFT) was taken into consideration to know about the dominant frequency by using MATLAB®. Frequency and amplitude values were plotted, and readable results were displayed on the computer screen, varying for each case depending on the eel’s distance from the bluff body in both $G_{x}$ and $G_{y}$. To observe the effect of varying the distance on energy harvesting, the flapping of the eel, frequency, and amplitude of the flapping eel were taken into consideration. The wake and vortex formation for each roughness condition were visualized in this experiment. Measurement accuracies were: velocity (±0.5% FS), voltage (±0.006% FSR, NI USB-6212), resistance (±1%, 1 MΩ Vishay), and amplitude (±1.2%, calibrated camera). Propagating these via the root-sum-square method yields a ± 0.37% uncertainty in power P = V_2_/R_opt_, resulting in a ± 4.1% uncertainty in the power coefficient C_p_ at 95% confidence. Through detailed analysis of 25 cases per roughness set, totalling 100 cases, the current study systematically investigated their effects. The findings provide details into the impact of surface roughness on the energy harvesting efficiency.

## Results & discussion

**Fig 4:**
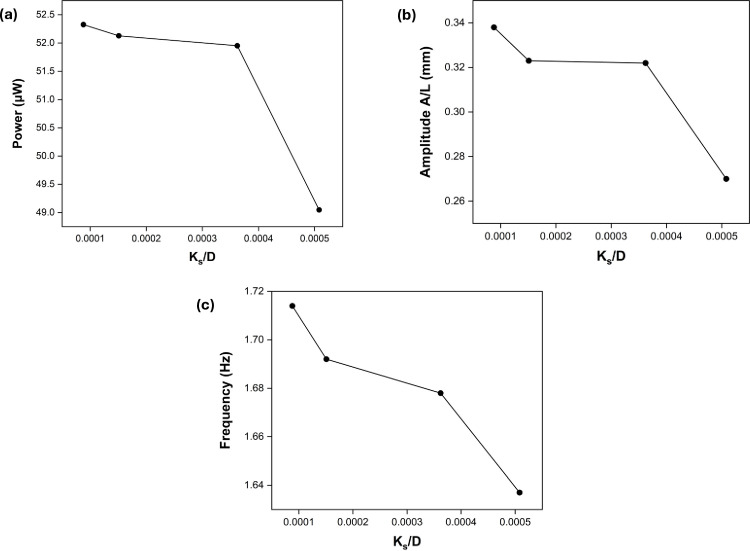
Plot of maximum power (a), amplitude (b) and frequency (c) of eel versus roughness of the cylinder.


From the experiments, the deduced plots of power (p), frequency (f), and amplitude (A/L) against the roughness values (K_s_/D) at $G_{x}$ = 1.25 and $G_{y}$ = 0, where the maximum energy was harvested for each surface roughness of the cylinder, referred to as the best efficiency power is shown in Fig 4. The best efficiency power ($P_{\text{eff }}$) expression is represented by Equation 1 where ρ is the fluid density, U is the free-stream velocity, S is the characteristic area (foil span × length), and $C_{P}$ is the power coefficient. It is noteworthy to observe that the power at the best efficiency decreased with an increase in the surface roughness of the cylinder. The best efficiency power correlated very well with the amplitude of the eel and its frequency, where both the amplitude and frequency increased with power. This is because the deflection of the beam is directly proportional to the strain in the beam, according to the Euler-Bernoulli equation (Equation 2), which in turn increases the power. The governing equation for the deflection w(x,t) of a flexible foil subjected to hydrodynamic forces. The parameters in the equation include the Young’s modulus of elasticity of the foil material (E), the second moment of area (I), the foil material density ($\rho_{s}$), thickness (h) and the structural damping coefficient ($c_{d}$). The term $F_{y}(x,t)$ represents the hydrodynamic lift force acting on the foil due to vortex shedding from the upstream cylinder.

$$P_{\text{eff }}=\frac{1}{2}\rho U^{3}SC_{P}$$ (1)

$$EI\frac{\partial^{4}w}{\partial x^{4}}+\rho_{s}h\frac{\partial^{2}w}{\partial t^{2}}+c_{d}\frac{\partial w}{\partial t}=F_{y}(x,t)$$ (2)

The strain energy ($U_{s}$) stored in the foil is proportional to the curvature and can be expressed as:


$$\epsilon (x,t)=\frac{\partial^{2}w}{\partial x^{2}}$$ (3)

$$U_{s}=\frac{1}{2}EI\int_{0}^{L}\;{\left(\frac{\partial^{2}w}{\partial x^{2}}\right)}^{2}dx$$ (4)

Since strain energy is directly linked to power output, higher foil deflection due to stronger vortex-induced forces leads to greater power generation. However, increasing the surface roughness of the upstream cylinder can disrupt vortex shedding, reducing the lift force amplitude and, consequently, the oscillation amplitude of the foil. This results in a decrease in the best efficiency power output. Additionally, it is important to highlight that the best efficiency power is maximum for a smooth cylinder (K_s_/D = $8.8\times 10^{-5}$); it decreased almost linearly at a low rate till K_s_/D = $3.62\times 10^{-4}$, after which it decreased significantly till K_s_/D = $5.08\times 10^{-4}$. Furthermore, the power varied from 52.3 μW at K_s_/D = $8.8\times 10^{-5}$ to around 49 μW at K_s_/D = $5.08\times 10^{-4}$ and the amplitude also decreased with an increase in the roughness of the cylinder, as shown in Fig 4(b). The maximum amplitude of around 0.338 was observed at K_s_/D = $8.8\times 10^{-5}$. However, the amplitude dropped to around 0.323 as K_s_/D increased to $1.51\times 10^{-4}$, followed by a very small change in amplitude till K_s_/D = $3.62\times 10^{-4}$. The effect of the roughness of the cylinder was maximum at K_s_/D = $5.08\times 10^{-4}$, where the amplitude dropped significantly to 0.27. It was observed that the frequency decreased at a higher rate for the entire range of roughness of the cylinder, as evident from Fig 4(c). The strong vortex shedding from the cylinder exerted the maximum force on it, due to which maximum power was extracted at $G_{x}$ = 1.25 and $G_{y}$ = 0. In addition to that, it was also observed that the maximum power at all the roughness values was independent of $G_{x}$ and $G_{y}$, since it always occurred at $G_{x}$ = 1.25 and $G_{y}$ = 0.

**Fig 5:**
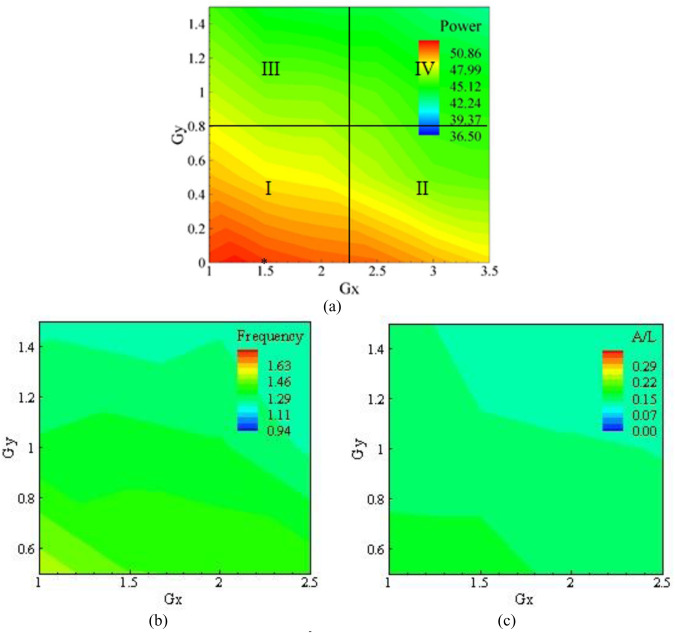
Results for K_s_/D  = 8.8 × 10^−5^, (a) Power, (b) Frequency, and (c) Amplitude.

**Fig 6:**
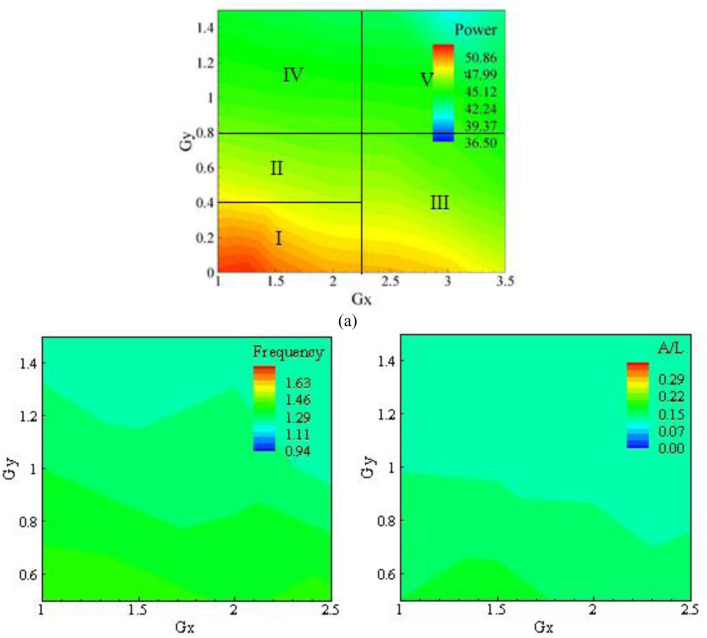
Results for K_s_/D  = 1.51 × 10^−4^ (a) Power, (b) Frequency, and (c) Amplitude.

Besides, the contours of power, amplitude and frequency on $G_{x}$ and $G_{y}$ plane for K_s_/D = $8.8\times 10^{-5}$, which is very small and can be approximated as a smooth cylinder, are presented in Fig 5. The power, amplitude, and frequency decreased with the increase in both $G_{x}$ and $G_{y}$ and it can be correlated to the vortex shedding in the wake of the cylinder. As the $G_{x}$ was increased, the vortices shed from the cylinder began to dissipate. This caused the strength of the vortex shedding to decrease, reducing the force on the eel and the amplitude in the x-direction. Likewise, increasing $G_{y}$ caused the flow to become axi-symmetric upstream of the eel, which reduced the force and thus the amplitude consequently. The frequency of the eel also decreased with the increase in $G_{x}$ and $G_{y}$ and it can be directly related to the vortex shedding behind the cylinder. The value of $G_{x}$ and $G_{y}$ corresponding to the best efficiency power is also shown in Fig 5(a) with an asterisk, which occurred at $G_{x}$ = 1.25 and $G_{y}$ = 0. In Fig 5(a), which is further divided into four regions, the variation of power was strong in region I, where the maximum extracted power was observed. The energy harvested decreased in region II and declined further in region III. The minimum power was extracted in region IV. By comparing Fig 5(a)–(c), it was observed that the frequency and amplitude varied with $G_{x}$ and $G_{y}$ significantly as compared to power. As the roughness of the cylinder increased, the power harvested from the cylinder decreased as illustrated in Fig 6, which shows the contours of power, amplitude and frequency on a $G_{x}$ and $G_{y}$plane for K_s_/D = $1.51\times 10^{-4}$.

The physical appearance of the cylinders and the increase in grain size or grit size were carefully examined to determine the point at which the cylinder was positioned just in front of the eel to produce maximum flapping. This was due to the maximum shear layer generation from both sides of the bluff body. The area in which the optimum point lay was determined after experimentation. The red color of the region near the origin verifies the maximum power harvesting at that point. The gap in the $G_{x}$ direction was 7 points, and the gap difference offset in the $G_{y}$ direction was 4 points. The experimentation on gap $G_{x}$ = 1.25 and $G_{y}$ = 0 was conducted to determine the peak value of power at the optimum point. The dimensionless distance is $G_{x}$ = S/D, where S was the distance from the center of the bluff body to the eel, and D was the diameter of the cylinder.

Moving away from the origin along the X-axis, the power generated by the bluff body decreased. The minimum power shown on the plot was measured to be 36.4 microwatts, while the maximum power observed was 52.325 microwatts. The fluctuation in the movement was discussed within this range, as it was a function of power harvesting. However, as one moves along the Y-axis, the flapping decreases abruptly due to the decreasing effect of the shear layer on the eel. This can be observed from the decreasing power shown by different colors. The red color shows the maximum power, followed by orangish-yellow, yellow, green, and bluish color, which showed the minimum flapping and reduced energy. This change in power generation was due to the increased surface texture and grain size, which alters the physics of the shear layer. The abrupt change in power along the Y-axis was caused by an increase in the span-wise gap between the bluff body and the eel. As the gap increased, the impact of the shear layer on the eel decreased as well, resulting in a smaller change in power generation. The bluish area dominates when the surface roughness reached 13.97 microns. The detailed investigation of the results was obtained from different dimensionless roughness factors used in the experimentation of artificially designed ranges of roughness. These results depict the effects of corrosion pitting on the aluminum cylinder during aging. Chemical reactions occur during the aging process, and the flow of salty water containing Na^+^ and Cl^-^ ions tends to corrode and remove the upper layer of material with the flow direction. The upper layer becomes weaker and is finally removed, revealing hollow pits, and eroding the surface. The reference point origin was marked on the bluff body, which was fixed in the fixture. The eel was moved from point to point to read the impact of the gap and the increase in the roughness factor.

Currently, the roughness of pits is correlated with energy gain, which becomes less efficient due to lack of smoothness. As a result, the flow passage is disturbed, and the wake region becomes narrower as roughness increases. The results of power, amplitude, and frequency are shown in Fig 6(a)–(c), respectively for K_s_/D = 1.51 × 10^−4^. The power is maximum at $G_{x}$ = 1.25 and $G_{y}$ = 0. The shear layer formed from the bluff body wake in both directions is maximum at this point and merges in such a way that the flapping area and frequency are also maximum, resulting in maximum power gain, as was previously shown in Fig 5. The roughness pitting of the cylinder tends to increase with aging, resulting in decreased efficiency to provide energy as a bluff body. This was confirmed by tests where the roughness was enhanced to 1.51 × 10^−4^, resulting in a decrease in the peak area of energy due to lesser flapping of eel in that area as the gap of $G_{x}$ and $G_{y}$ were varied in a constant manner of 0.5 mm. As the eel is moved away from the origin in the x-direction, the spanwise gap from the bluff body increases, and the impact of vortex energy evolved from the bluff body wake is not able to reach the same level as before, leading to less flapping and reduced power. The optimal value of power for $G_{x}$ and $G_{y}$ is (1.25, 0), as shown in Fig 6.

**Fig 7:**
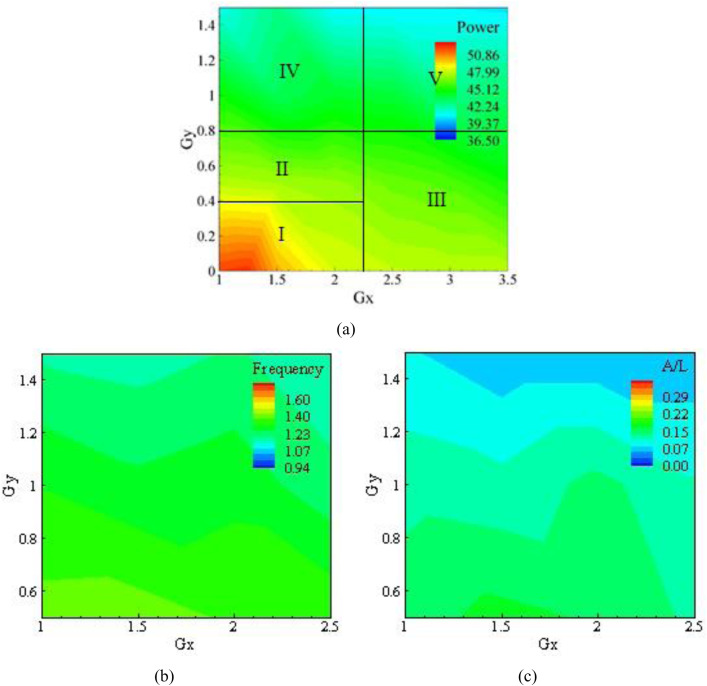
Results for K_s_/D  = 3.62 × 10^−4^, (a) Power, (b) Frequency, and (c) Amplitude.

In addition to the above, Fig 7 illustrates the contours of power, amplitude, and frequency at a roughness of K_s_/D = 3.62 × 10^−4^. The graphs depicting the reduction of power, frequency, and amplitude are shown in bluish color, while the reddish color shows the maximum power of approximately 52.325 microwatts. The minimum power harvested is shown in dark blue and is 36.4 microwatts. Point (1, 0) is taken as the origin for the span-wise gap in the $G_{x}$ and $G_{y}$ directions. The optimum location of power in $G_{x}$ and $G_{y}$is (1.25, 0), as shown in Fig 7.

**Fig 8:**
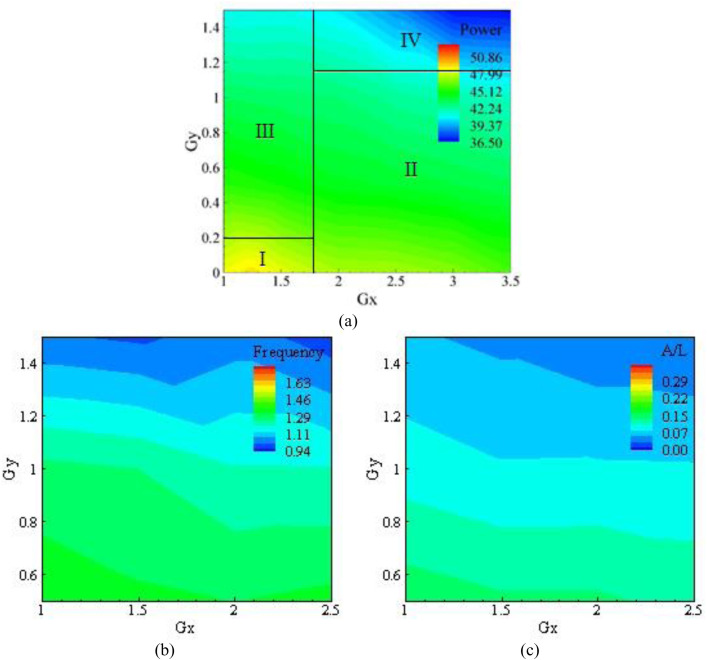
Results for K_s_/D  = 5.08 × 10^−4^, (a) Power, (b) Frequency, and (c) Amplitude.

Upon deep observation of the plots at a roughness of $5.08\times 10^{-4}$, it was clear that the power, frequency, and amplitude were at their minimum, as indicated by the dominant greenish and bluish colors in the graph. A small region with orange and yellow color near the origin (1, 0) is visible as the optimum power location in the $G_{x}$ and $G_{y}$ direction is found to be (1.25, 0). The blue-colored region away from the optimum point is a low-energy region, as observed in the experimentation, where the impact of the flapping eel is low, and flapping is almost negligible. Additionally, the amplitude is much less than the optimal point values. The least amount of energy is depicted in the bluish region at the point (3, 1.5), while the second minimum energy point is (3.5, 1.5), as shown in Fig 8.

## Generation of maximum output

**Fig 9:**
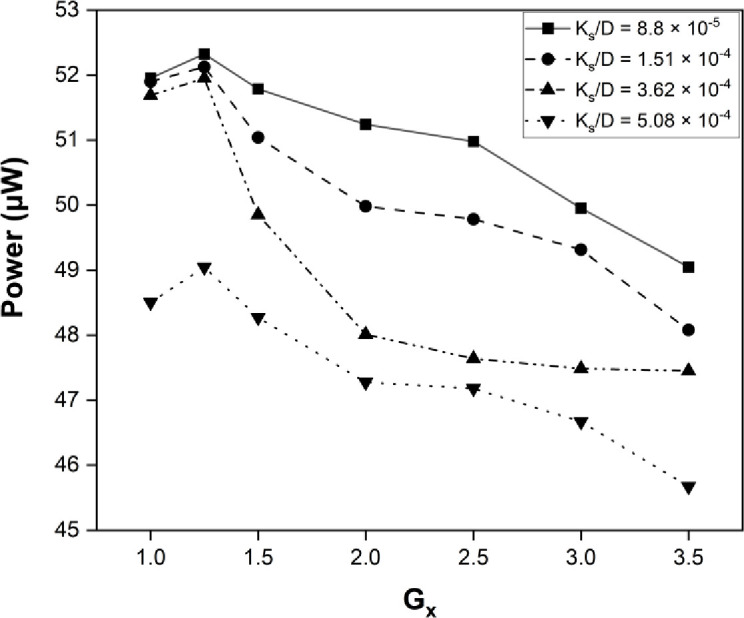
Graph for maximum Power at G_y_ = 0.

**Fig 10:**
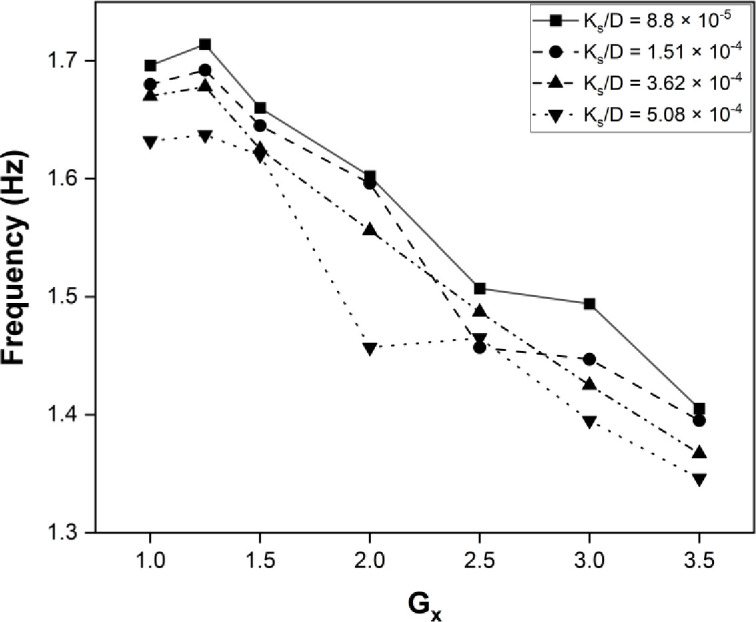
Graph for maximum Frequency at G_y_ = 0.

**Fig 11:**
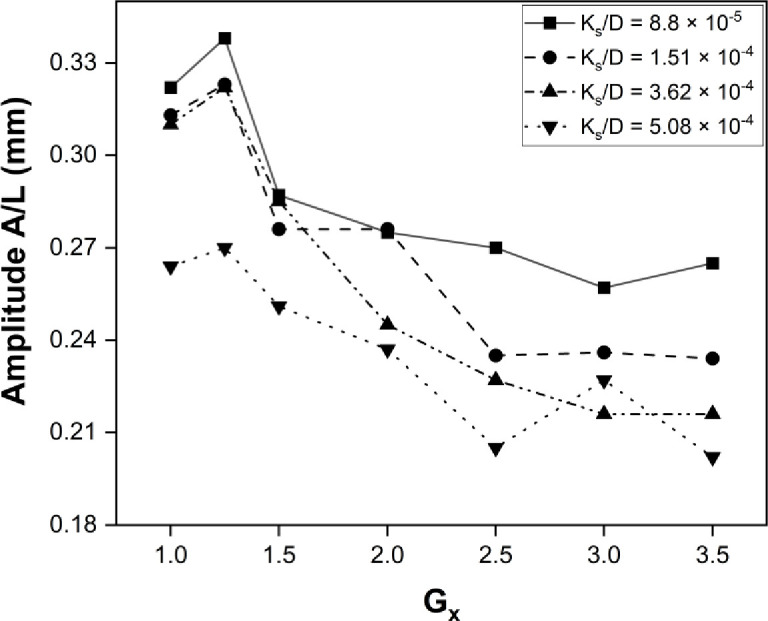
Graph for Max Amplitude/Length at G_y_ = 0.

The maximum power harvested was obtained at $G_{x}$ = 0, while keeping the $G_{y}$ gap at 1.25, which was found to be the optimal point for energy harvesting from the cylinder. The roughness of the cylinder varied from the maximum to minimum range, as illustrated in the plot shown in Fig 9. The dimensionless roughness factor was different for each case. The shear layer narrows as the roughness factor increases, and energy dissipates earlier with an increase in roughness. The effectiveness of the shear layer and its dissipation also decreased as the roughness factor increased. Also, the frequency graph illustrates the maximum frequency obtained, with the optimum point marked as (1.25, 0) in Fig 10 The frequency is maximum for the smooth bluff body and at a point close to the bluff body. Moving away in $G_{x}$ and $G_{y}$, the response of the eel decreases due to the dissipation of the shear layer. The A/L ratio of the flapping eel depends on the surface roughness and the optimal point where the maximum power is located, which ultimately determines the amplitude and frequency of flapping, resulting in the harvested power. The maximum amplitude observed at point (1.25, 0) for K_s_/D = $8.8\times 10^{-5}$ is plotted in Fig 11.

**Fig 12:**
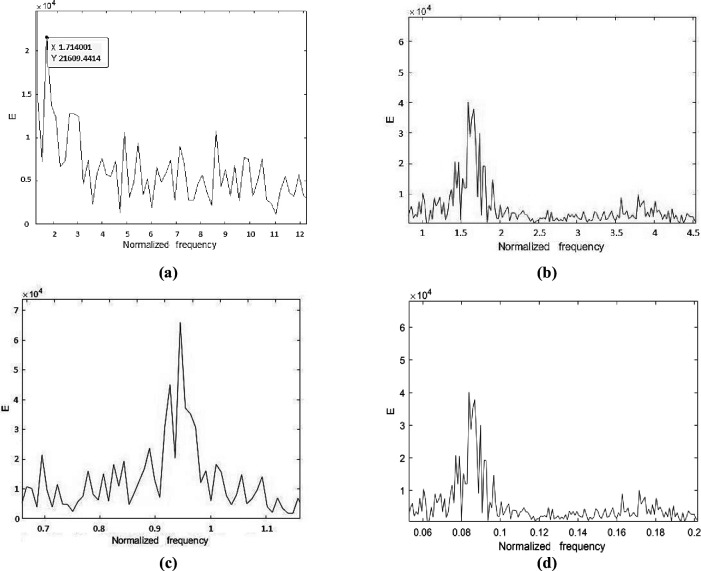
Frequency Chart for optimal maxima case (Frequency = 1.714) (a), second optimal maxima case (Frequency = 1.692) (b), optimal minima case (Frequency = 0.946) (c) and second optimal minima case (Frequency = 0.957) (d).

**Fig 13:**
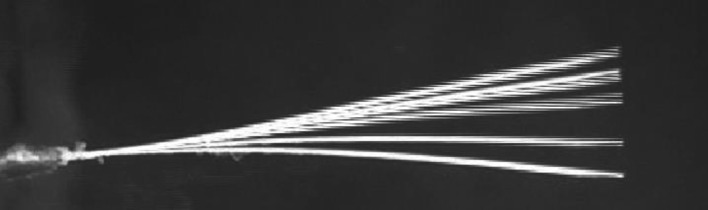
Amplitude for A/L = 0.29.

**Fig 14:**
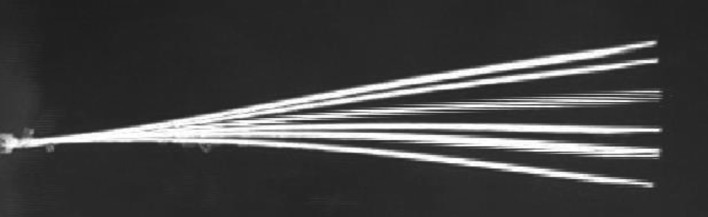
Amplitude for A/L = 0.338.

**Fig 15:**
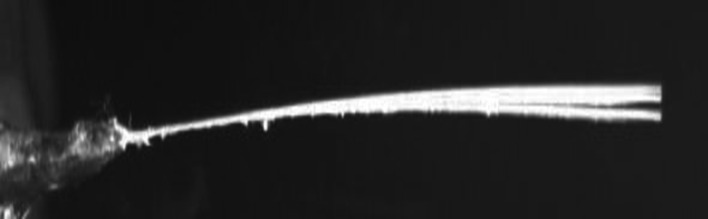
Amplitude for A/L = 0.02.

**Fig 16:**
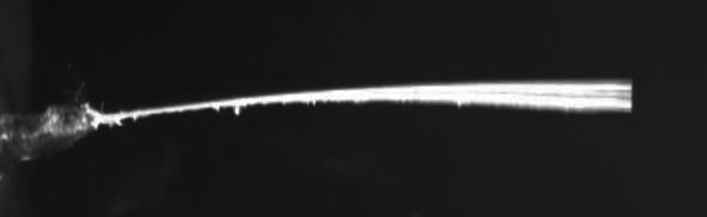
Amplitude for A/L = 0.03.

The cylinder with a roughness of $8.8\times 10^{-5}$ exhibited the maximum normalized frequency of 1.714 at the point of experimentation (1.25, 0), which is also the optimal point. This observation is shown in Fig 12(a). The second-highest normalized frequency of 1.692 was observed at the cylinder with a roughness of $1.51\times 10^{-4}$ at the same point (1.25, 0), which is depicted in Fig 12(b). The minimum values for normalized frequencies are shown in Fig 12(c) and (d). The minimum values occur at the points (3.5, 1.5) and (3, 1.5), with corresponding minimum frequencies of 0.946 and 0.957, respectively. Moreover, Figs 13–16 illustrate the flapping of eel corresponding to A/L ratio of 0.29, 0.338, 0.02 and 0.03, respectively.

Table 3 lists the summary of the key results obtained from the current study. The results indicate a clear reduction in power, frequency, and amplitude as surface roughness increases. The smooth cylinder, which serves as the reference case, produces a power output of 52.325 µW, a vortex shedding frequency of 1.6955 Hz, and a non-dimensional amplitude of 0.32176 A/L. As roughness increases, all three parameters experience progressive reductions. At the highest roughness level (K_s_/D = $5.08\times 10^{-4}$), power output decreases by 6.26%, frequency drops by 4.50%, and amplitude sees the most significant reduction of 20%. These reductions suggest that increasing surface roughness disrupts the vortex shedding process, weakening vortex formation and reducing oscillatory energy. This effect is mainly due to increased turbulence and altered boundary layer behavior, which interferes with the organized shedding of vortices. The decline in power output indicates that rougher surfaces may limit the efficiency of energy harvesting systems that rely on vortex-induced vibrations.

**Table 3:** Summary of experimentation results.

Non-dimensional Roughness (Ks/D)	Power (µW)	Frequency (Hz)	Amplitude (A/L)
8.8 × 10 ⁻ ⁵ (Smooth Surface)	52.325	1.6955	0.32176
1.51 × 10 ⁻ ⁴	↓ 1.35%	↓ 1.18%	↓ 5.96%
3.62 × 10 ⁻ ⁴	↓ 3.46%	↓ 2.42%	↓ 11.56%
5.08 × 10 ⁻ ⁴	↓ 6.26%	↓ 4.50%	↓ 20.00%

## Conclusion

This study experimentally examined the influence of surface roughness, representing material aging, on the performance of piezoelectric eel-based energy harvesters placed downstream of a cylindrical bluff body. Results showed that increased roughness significantly reduced power output, flapping frequency, and amplitude. The smoothest cylinder (K_s_/D = $8.8\times 10^{-5}$) achieved the highest power generation of 52.325 µW, while the roughest (K_s_/D = $5.08\times 10^{-4}$) dropped to 36.4 µW, marking a 6.26% decline in power output. Flapping frequency and amplitude also decreased by 4.5% and 20%, respectively. The optimal energy harvesting occurred at $G_{x}$ = 1.25, $G_{y}$ = 0, corresponding to maximum flapping behavior. Furthermore, increased roughness narrowed the lock-in region critical for effective VIV-based harvesting. These findings confirm that surface aging negatively impacts energy harvesting efficiency and underscore the importance of maintaining bluff body surface conditions in real-world marine environments to ensure long-term harvester performance. Future research could extend this work by examining a broader range of Reynolds numbers and flow velocities to better reflect real-world conditions. The impact of non-uniform surface roughness, biofouling, and sediment deposition should be investigated to simulate natural aging and degradation of marine structures. Computational simulations alongside experimental studies can provide better understanding of vortex dynamics and piezoelectric behavior. Additionally, exploring alternative piezoelectric materials, multi-layered structures, and multi-degree-of-freedom configurations may lead to more efficient and durable energy harvesting systems for aquatic environments.
